# A Few Cases Cured with the High and Highest Potencies

**Published:** 1892-08

**Authors:** J. Emmons

**Affiliations:** Richmond, Ind.


### A FEW CASES CURED WITH THE HIGH AND HIGHEST POTENCIES.

J. Emmons, M. D., Richmond, Ind.

[Read before the Indiana Institute, May, 1892.]

Case 1.—Mr. M., aged thirty-five, dark skin, black hair, bilious temperament, had an attack of la grippe, followed by what his physicians (of whom he had five, four allopaths and one eclectic) called cerebro-spinal-meningitis with its complications. For six weeks they had been bombarding the enemy without relief. He told me his drug bill aside from his doctors' bill was $35. By some mysterious providence he was moved to call me.

Symptoms—Pain in the base of the brain, continuous but much worse *every evening*, so that convulsions and insensibility were the result occasionally. The only relief was from *tight band-*

*aging* and *cold applications*. He would cry and could not help it when the pains were the greatest. Diagnosis, neuralgia, prescription, Pulsatilla$^{cc}$, three doses three hours apart relieved him in twenty-four hours, and he made a good recovery without any more medicine.

CASE 2.—Mr. D., aged thirty, married, fair skin, light hair, etc. He, too, had been a victim of la grippe for six weeks, had a constant headache (neuralgic). Had taken large doses of Quinine, Antipyrine, and all the fool cures that doctors and some that were not advise, from which he received no benefit, but grew worse. Finally the spirits or some other influences induced him to send for me. I found him wringing his hands, pressing his head, swaying his body, and groaning with pain, pain continuous but much worse in paroxysms, pain *through* the *temples* from one to the *other*, sensation as if an iron bolt was passed from *side* to *side* with a nut on each end and was *being screwed up*, was *hungry* all the *time*. Phos.$^{cc}$, three doses three hours apart. Reported at my office in twenty-four hours free of pain, a thing that had not been for six weeks, a good recovery without more medicine. I have underscored the symptoms that led to the remedy.

